# Perspectives in Manipulating EVs for Therapeutic Applications: Focus on Cancer Treatment

**DOI:** 10.3390/ijms21134623

**Published:** 2020-06-29

**Authors:** Katarzyna Nazimek, Krzysztof Bryniarski

**Affiliations:** Department of Immunology, Faculty of Medicine, Jagiellonian University Medical College, 31-121 Krakow, Poland; mmbrynia@cyf-kr.edu.pl

**Keywords:** anti-oncomiRs, anti-tumor immune response, anti-tumor therapy, drug delivery, extracellular vesicles, exosomes, immune regulation, miRNA

## Abstract

Extracellular vesicles (EVs) receive special attention from oncologists due to their assumed usefulness as prognostic markers, vaccines to induce anti-cancer immune response, and physiological delivery tools. The latter application, which supports the reduction of side effects of treatment, is still fraught with many challenges, including established methods for loading EVs with selected cargo and directing them towards target cells. EVs could be loaded with selected cargo either in vitro using several physicochemical techniques, or in vivo by modification of parental cell, which may have an advantage over in vitro procedures, since some of them significantly influence EVs’ properties. Otherwise, our research findings suggest that EVs could be passively supplemented with micro RNAs (miRNAs) or miRNA antagonists to induce expected biological effect. Furthermore, our observations imply that antigen-specific antibody light chains could coat the surface of EVs to increase the specificity of cell targeting. Finally, the route of EVs’ administration also determines their bioavailability and eventually induced therapeutic effect. Besides, EV membrane lipids may possibly possess immune adjuvant activity. The review summarizes the current knowledge on the possibilities to manipulate EVs to use them as a delivery tool, with the special emphasis on anti-cancer therapy.

## 1. Introduction

Cells of multicellular organisms can communicate with one another through the release of extracellular vesicles (EVs). They are present in virtually all body fluids and can freely circulate between tissues and organs, even crossing the barriers. Thus, EVs mediate intercellular signaling in an auto-, para-, and endocrine manner and induce the eventual biological effect in acceptor cell by receptor–ligand interactions and by delivery of regulatory cargo. In this regard, EVs provide a platform for an exchange of cell-derived constituents, including nucleic acids, proteins, lipids, and metabolites, which physiologically contributes to maintenance of homeostasis. It is worth noting that EVs are multidirectional immune modulators. Accordingly, recent report revealed that the same EV subtype that target antigen-presenting cells can regulate both humoral and cell-mediated immune responses [1].

Consequently, dysregulation of EV-mediated communication drives pathological processes. Simultaneously, substantial changes in cellular origin, composition, and function of EVs are observed under the pathological conditions. This fact made EVs a promising candidate for using as biomarkers of various disorders, including carcinogenesis. Furthermore, growing awareness of factors determining EVs’ biogenesis and their eventual function greatly supports the attempts to manipulate EVs for therapeutic application as vaccines and delivery tools. However, inclusion of EV-based therapeutics in clinical practice still requires extensive studies and solving many problems.

This review summarizes the current research findings and discusses the future perspectives in manipulating EVs for further usage as a delivery tool, with the special focus on anti-cancer therapy.

## 2. Proposed Clinical Applications of EVs in Oncology

Recently, EVs have received special attention from oncologists both due to their physiological capability to deliver signaling molecules and their involvement in carcinogenesis. In addition, EVs play an important role in regulation of innate and adaptive immunity, including anti-tumor responses. Thus, EVs are considered promising tools for diagnostic and therapeutic purposes [2,3,4]. So far, studies on the clinical applicability of EVs proposed their usefulness as prognostic biomarkers, vaccines to induce anti-tumor immune responses, and drug delivery tools [5]. However, due to the limited knowledge on complex EVs’ biogenesis and cargo sorting, clinical applications of EVs are still fraught with many challenges.

### 2.1. EVs as Cancer Biomarkers

In most cases, diagnostic process in patients with suspected cancer involves histopathological examination. Recently, liquid biopsies have been proposed for early monitoring and outcome prediction in oncological patients to avoid the invasive procedures, especially in the case of solid tumors. Soon after introducing them to clinical practice, EVs and their cargo became considered promising biomarkers of cancer development and progression for “real-time monitoring”.

Accordingly, cells of each tissue microenvironment can release EVs, which may then freely circulate in various body fluids. In addition, abundant populations of EVs generated by each parental cell change constantly, depending on the current condition of the cell, which is determined by many factors, including activation state, metabolism, senescence, and recently received signals. This, in turn, is responsible for the tremendous heterogeneity of EV populations obtained in biological samples. However, each change in EV population seems to reflect a change in tissue functioning. Thus, research attempts to evaluate EVs’ biomarker function seek to establish a pattern that would be characteristic for a particular circumstance. Assessment of an EV pattern includes testing of surface markers’ expression and enclosed cargo, which are changing in time. This feature allows the “real-time monitoring” of a tumor [6].

EVs’ testing would greatly advance personalization of cancer diagnosis. However, the use of EVs as biomarkers still requires creating of standardized protocols for liquid biopsy collection, sample processing for EVs’ separation, and isolation of EV-enclosed cargo.

#### 2.1.1. EV-Surface Molecules as Predictors for Patient Responsiveness to Therapy 

Checkpoint inhibitor therapies, including blockage of programmed death receptor 1 (PD-1)-programmed death-ligand 1 (PD-L1) interaction with the use of monoclonal antibodies, receive special attention from oncologists. However, patient response rate to anti-PD-1 treatment is rather low. Recent research findings revealed the correlation of the level of PD-L1 on circulating EVs in melanoma patients with their response to treatment, i.e., a higher level of circulating EV-carried PD-L1 prior to the treatment predicted the poorer clinical outcome [7]. This study also suggested that the failure of anti-PD-1 therapy may result from both dynamic nature of PD-L1 expression by tumor cells and possible binding of PD-1 on cytotoxic T cells by EV-derived PD-L1. The latter may cause T cell “exhaustion”. Thus, expression of PD-L1 on circulating EVs was proposed a predictor for patient responsiveness to PD-1 inhibitor therapy [7]. In addition, PD-L1 expression could be used to confirm that assayed EVs are of tumor origin [8].

#### 2.1.2. EV-Expressed Micro RNAs (miRNAs) as Predictors of Drug Resistance and Metastatic Potential

Research findings suggest that EV-carried, oncogenic miRNAs may confer cancer resistance to chemotherapies by affecting immune cells in the tumor niche. Accordingly, ovarian cancer-derived EVs have been shown to deliver miRNA-1246 to tumor-infiltrating M2 macrophages, which reduces the synthesis of Cav1 protein. Consequently, M2 macrophages could synthesize the multi drug resistance (MDR)-1 protein, which promotes their pro-oncogenic activity [9]. On the other hand, recent studies identified several miRNAs, including miR-9, miR-10b, and miR-182, responsible for up-regulation of metastasis [10]. Enclosing the pro-metastatic miRNAs into EVs supports invasion of neighboring cells, which promotes tumorigenesis [10]. Hence, testing of miRNA profile in patients’ EVs may constitute a marker to predict tumor resistance to treatment and metastatic potential.

### 2.2. EVs as Vaccines Inducing Anti-Tumor Immune Responses

Tumor tissues are infiltrated by different populations of immune cells. However, they are triggered by tumor-derived signaling molecules, such as cytokines, immune checkpoint stimulators, and microvesicles, to mediate immune tolerance. This allows tumor cell escape from detection and cytotoxic killing [11]. Thus, breaking of immune tolerance to tumor is an important determinant of cancer immunotherapy success. Targeting of dendritic cells (DCs) is considered a promising to induce anti-tumor immune responses [12].

Modified DCs have been tested in clinical trials as anti-cancer vaccines for over twenty five years [13,14]. In brief, isolated DCs are cultured in vitro and either loaded with tumor-derived antigens or genetically manipulated to become immunogenic. After maturation, modified DCs are transferred to patients to induce specific T cell responses against tumor cells [13]. However, DCs are highly sensitive to changes in microenvironmental conditions [14]. Consequently, their immunogenic activity in vivo can be impaired by tumor-derived signaling molecules. This in turn may be responsible for the negative clinical outcome of DC-based immunotherapy.

At present, some research attempts focus on improving the methods of DC modification [13]. Instead, other studies proposed to use EVs secreted by modified DCs as anti-tumor vaccines [15]. DC- and EV-based immunotherapies share similar challenging features, but EVs have a significant advantage over DCs. Their activity seems to be stable in changing ambient conditions [15].

#### 2.2.1. DC-Derived EVs

Small EVs derived from modified DCs, often called “Dexosomes” or “Dex”, have proved their therapeutic potential in several preclinical studies [16]. Thus, they have been subjected to phase I and II clinical trials in patients with advanced malignancies, including inoperable non-small cell lung cancer [17,18], and metastatic melanoma [19], as recently reviewed [15]. Accordingly, these clinical trials confirmed the safety and feasibility of “Dex”-based therapies, and revealed the propensity of “Dex” to induce anti-tumor immune responses [15]. However, much more observations from clinical trials are needed to ultimately estimate their therapeutic effects.

#### 2.2.2. Tumor-Derived EVs

On the other hand, cancer cell-derived EVs are considered a source of tumor antigens for DC priming. So far, several studies have tested their immunogenic potency in animal models [20,21]. Tumor-derived EVs loaded in vitro with immunostimulatory miRNAs were found to activate maturation of DCs, which suggested their usefulness as DC-targeting vaccines [22].

#### 2.2.3. Engineered Nanovesicles

Experimental attempts have been undertaken to construct synthetic DC-targeting nanovesicles, which would stimulate DCs for presentation of tumor antigens to cytotoxic T cells in vivo [23]. Interestingly, recent report revealed that administration of EVs together with a lipid adjuvant increased the cross-presentation of EV-derived tumor antigens by DCs, which allowed for induction of CD8+ cytotoxic T cells [24]. Accordingly, one can speculate that, in certain circumstances, lipids composing EV membrane may act as immune adjuvants to increase the immunogenicity of EV-contained antigens.

Obviously, engineered nanovesicles can also target other immune cells to train them how to fight against cancer. A recent review by Kroll et al. has summarized the principles, advantages, and challenges of engineering of biomimetic nanovesicle-based vaccines for cancer therapy [25].

### 2.3. EVs as a Delivery Tool

EVs are physiologically produced by virtually all body cells and, thus, are abundant in body fluids, from which they can be isolated. Their bioavailability is increased by the ability to cross biological barriers. As they are considered biocompatible, EVs could be safely administered not only to their donor, but also to other organisms of the same species. Overall, their biological characteristics drives the attempts to use EVs as a therapeutic tool for delivery of drugs, including chemotherapeutics, toxins, miRNAs, anti-miRNAs, and anti-oncomiRs [26]. EVs have already been tested as physiological anti-cancer drug-delivery platforms in several in vitro studies or animal models [27,28]. However, their clinical applications are fraught with many challenges. As discussed below, those include standardization of EVs’ isolation methods, techniques for selective cargo loading, strategies for specific cell targeting, and administration protocols.

#### EVs for Drug Delivery

Despite of the above concerns, six patients with end-stage lung cancer have been administered with cisplatin-loaded vesicles released by the human lung carcinoma cell line. Results suggested that enclosing of the drug with EVs may reverse multidrug resistance developed by patients after several rounds of chemotherapy [29].

Enclosing drugs in cell-targeted EVs would greatly increase their stability and limit systemic adverse effects. Furthermore, it may also intensify the therapeutic effect by increasing the number of drug molecules derived to each target cell. This could be additionally strengthened by packaging drug-loaded nanoparticles into EVs [30]. Besides, such drug modifications can also support its incorporation into EVs [31] and allow tracking of EV accumulation [32]. The latter enables theranostic application of EVs carrying modified drug molecules [31]. Similarly, labelling EVs, for instance with radioisotopes, has a great implication for their theranostic application [33].

Altogether, EVs are proposed to deliver various drugs to induce stable and long-lasting therapeutic effects. However, their inclusion in clinical practice requires extensive studies to overcome all challenges.

## 3. Perspectives in Manipulating EVs for Therapeutic Applications

EV-based delivery systems are promising candidates to significantly improve the efficacy of cancer therapies. However, their future clinical application requires standardization of the following aspects: (i) EVs’ generation and isolation methods, (ii) techniques for selective cargo loading, (iii) strategies for specific cell targeting, and (iv) protocols of their administration, including doses, routes, and timing.

### 3.1. EVs’ Isolation Methods

Methods most commonly used for isolation of EVs have recently been comprehensively reviewed and discussed in the terms of their impact on biological function of EVs [34]. However, there is still a long way to develop a unified protocol for the isolation of a particular EV subtype from a particular biological material. One of the promising approaches to isolate EVs from any biological fluid is based upon size-exclusion chromatography that provides high purity of EV isolate [35,36,37].

In multicellular organisms, EVs are a part of physiological communication system. Therefore, they seem to be much more biocompatible than artificially produced vesicles and liposomes. However, EV populations isolated from body fluids by ultracentrifugation are largely heterogeneous. This prompted researchers to develop the procedures of in vitro stimulation of cultured cells to release EVs. However, cell-released EVs from ultracentrifuged supernatants were found to be heterogeneous as well [38]. Thus, EVs’ isolation from both body fluids and cell culture supernatants requires much more precise methods for separation of a particular EV subtype. Those include magnetic bead-based immunoaffinity method [39] and antigen affinity chromatography [40]. By using specific antibodies, both methods should allow the isolation of EVs expressing particular antigen of choice. Otherwise, the use of antigen-coated beads or polymers for chromatography separates EVs that are able to bind the antigen, for example, due to the surface expression of antigen-specific antibody light chains (LCs) [40]. However, the process of EVs’ elution from chromatographic columns or their detaching from the magnetic beads may influence their physical and chemical properties and, consequently, their biological activities. Thus, this aspect requires further investigation. Accordingly, our observations suggest that chromatographically-separated EVs eluted with acidic guanidine from column filled with Sepharose linked with either trinitrophenol (TNP) hapten [40], casein hydrolysate [41], or anti-CD9 monoclonal antibodies [41,42] preserved their biological activity.

### 3.2. Approaches to Loading EVs with Selected Cargo

One can assume that EV-contained cargo is a main determinant of the therapeutic efficacy of their application. As mention above, EVs may carry a great variety of biologically active molecules, including RNAs, proteins and lipids, which makes them a conveyor of virtually unlimited types of cargos. The cargo can be packed into EVs during their intracellular biogenesis or extracellularly after EVs’ exocytosis. The latter generally happens in in vitro conditions [43], but some results suggest the possibility of loading of freely circulating RNAs into EVs also in vivo [44]. Accordingly, the methods for loading EVs with selected molecules can be classified into two main groups, i.e., those based on modification of parental cells and those adapting physicochemical techniques enabling in vitro loading [45].

Parental cells can be passively loaded with chosen molecules. Along these lines, human gingival mesenchymal stromal cells were shown to uptake the chemotherapeutic drugs during standard cell culture [46]. Furthermore, one of these drugs, namely paclitaxel, was then found in cell-secreted EVs that expressed anti-cancer activity in vitro [47]. Similar activity was observed in the case of paclitaxel-carrying EVs released by mouse mesenchymal stromal cells [48]. In addition, mouse and human tumor cell lines were also shown to release drug-containing EVs after simple culturing in the presence of different chemotherapeutics [49]. In such cases, one can speculate that the drug is passively packaged into EVs during their formation. On the other hand, EV-parental cells can be transfected or transduced by non-viral or viral vectors, respectively, to produce the encoded molecules. These would likely be then actively sorted into EVs during their biogenesis. Accordingly, parental cells transfected with different plasmids were shown to secrete EVs that contained the plasmid-encoded products, including antibody protein and mRNA for enzyme that activates the chemotherapeutic prodrug. As a result, EVs were able to deliver mRNA to the cells of HER2-positive human breast tumor xenografts in a targeted manner due to the surface-expressed anti-HER2 antibody, which inhibited the growth of the xenografts in mice [50]. Interestingly, the later results suggested that EVs may deliver in vitro transcribed enzyme-encoding mRNA, which allows to eliminate the potentially harmful plasmid transfection of EV-parental cells [51]. Another interesting possibility was proposed by Sancho-Albero et al. [31]. The authors reported that hollow gold nanoparticles incubated with EV-parental cells are much more efficiently incorporated into EVs after their PEGylation. Delivery by EVs may greatly improve the accumulation of PEGylated gold nanoparticles in tumors [31]. However, many variables have to be taken into account while using these strategies. Therefore, at present, much more commonly used strategies are based on loading EVs with selected cargo after their isolation [45]. Maintaining the EVs’ integrity, allowing to protect the incorporated cargo from extracellular degradation or inactivation, is one of the features that should be considered while choosing the loading method. For these purposes, various methods are used, including passive loading, electroporation, treatment with saponins, dialysis, freeze-thaw cycles, sonication, and extrusion (Table 1).

#### 3.2.1. Passive Loading

Passive loading of EVs with particular molecules involves their joint incubation, most commonly at room temperature and in standard culture medium or in phosphate buffered saline (PBS) [52]. In some cases, prior to mixing with EVs, molecules are dissolved in special media or buffers, such as those dedicated for electroporation [53]. This is assumed to facilitate their loading. The time of incubation is usually established by each research group individually, but most commonly does not exceed 20 min. However, a simple protocol of passive loading of small molecules, including drugs and small interfering RNAs (siRNAs), has already been proposed [54]. Interestingly, in this protocol, the efficacy of rhodamine B incorporation into EVs was slightly higher, when compared to its loading supported by electroporation [54]. Other studies attempting to load various porphyrins revealed that the efficacy of passive loading increases with the hydrophobicity of molecules, i.e., the highest efficacy could be reached for the most hydrophobic compound [53]. This observation was also confirmed in the case of curcumin. Its loading into EVs from mouse lymphoma EL-4 cells increased curcumin’s bioavailability and anti-inflammatory activity in mouse model of septic shock [55]. Similarly, loading of catalase into EVs from RAW 264.7 macrophage cell line enhanced its stability and protective activity against oxidative stress in a mouse brain inflammation model. However, when comparing active and passive methods, the weakest effects were observed when catalase was loaded into EVs by passive incubation [56].

Likewise, siRNA passively incubated for 15 min at room temperature with EVs from HEK293T cells has then been barely detected in extensively washed and filtered EV preparations [54]. However, our studies seem to bring the contrary results. We have passively incubated for 30 min at 37 °C the mouse T cell- or B cell-derived EVs with nucleic acids, i.e., DNA/RNA extracted from EVs, RNAs purified from these extracts, synthetic miRNA-150 or synthetic anti-miRNA-150 molecules, which was followed by ultracentrifugation to remove the excessive RNA molecules. This loading strategy emerged sufficient to induce miRNA-150-mediated regulatory effects in immune effector cells treated with these miRNA-loaded EVs [40,44]. However, we assumed that a part of miRNA molecules was actually incorporated into EVs, while some adhered to EV surface. Thus, further studies are planned to evaluate the efficacy of miRNA incorporation. The discrepancies between the protocols regarding the efficacy of passive loading of RNAs into EVs may result from the differences in EVs’ purification after loading, since RNA molecules only adhered to EV surface may possibly be lost at this step [57]. Even though the use of passive loading is so far limited due to its an as yet unpredictable efficacy, it is a very promising strategy as it seems to have the least impact on EVs’ quality.

#### 3.2.2. Electroporation

Electroporation is a commonly used and highly efficient method for introduction of molecules, nucleic acids especially, into cells. It involves generation of electrical pulse that enables transient formation of pores in cell membrane, through which the charged molecules can freely pass into cytoplasm. Due to its simplicity and wide accessibility, researchers have begun to use electroporation method to actively load the cargo into EVs [26]. For anti-cancer therapy, experimental attempts showed the successful electroporation-induced loading of miRNAs into tumor cell-derived EVs [22] and into plasma-derived EVs [58], and of doxorubicin into EVs released by bone marrow-derived mesenchymal stem cells [59]. In addition, 5-fluorouracil and miR-21 inhibitor oligonucleotide were incorporated into EVs by electroporation to allow their targeted co-delivery to colon cancer cells, which may reverse drug resistance [60].

However, electric pulse may damage membrane integrity, and thus greatly impair EVs’ quantity, quality, and biological activity. Accordingly, when compared with other active loading strategies, electroporation is often less effective for molecules other than RNAs [31,61], while in the case of RNA, the efficacy seems to be significantly greater [54]. Therefore, the use of electroporation still requires standardization of the protocol, including optimization of pulse and field strength parameters, to balance the efficiency and EV damage ratio. Along these lines, Faruqu et al. [62] proposed a protocol for loading EVs with therapeutic siRNA by electroporation. The loading efficacy was estimated at 10–20%, and the efficacy of in vitro uptake of siRNA-loaded EVs by PANC-1 cancer cells at around 40%. In some instances, EVs were found to aggregate after electroporation, but this effect may likely be minimized by the use of specially prepared culture medium [63].

Notably, recent report uncovered the novel strategy enabling the large-scale generation of mRNA-encapsulating EVs by the process termed cellular nanoporation, combining EV-parental cell transfection and stimulation to release EVs [64]. Due to its expected very high efficiency, cellular nanoporation may be a breakthrough discovery enabling the widespread use of modified EVs in therapy.

To overcome the need to physically induce the electric potential across EV membrane, some chemical transfection methods have been proposed. Lipofection allows transient transfection of selected cargo into EVs in a reproducible manner, but the efficacy usually is inadequate [26]. Interestingly, siRNA encapsulated into milk whey EVs using lipofection was found stable in in vitro conditions simulating the digestive process [65]. Treatment with detergents is also considered for permeabilization of EV membrane.

#### 3.2.3. Treatment with Saponins

Simultaneous permeabilization of EV membrane was suggested to increase the efficacy of passive loading strategy. Then, it was classified as a separated active loading procedure. Saponins are currently the most widely used detergent for these purposes. Saponins are chemical compounds belonging to a group of amphipathic glycosides. They are plant-derived, natural soapy substances that can be used as non-ionic detergents. Additionally, saponins are a part of an aforementioned lipid adjuvant proposed to increase the cross-presentation of EV-derived tumor antigens [24].

So far, several studies employed saponin-based loading technique, showing its high efficacy [31,53,56,66,67,68]. This strategy likely preserves the integrity of EV membranes [45], and can be employed for encapsulating of molecules that are insensitive to detergents. On the contrary, saponins at higher concentrations may be used to release proteins and other compounds from EV membranes [69].

#### 3.2.4. Sonication

Sonication is a physical method that applies the sound energy to agitate particles in a preparation. It was firstly used to break up clumped EVs from blood plasma [70]. Then, this method was found to greatly facilitate the incorporation of various molecules into EVs during their co-incubation, supposedly by reducing the rigidity of EV membranes [71]. One of the first studies using sonication for loading of catalase into RAW 264.7 macrophage-derived EVs showed its high efficiency, comparable with extrusion and saponin treatment [56].

As mentioned above, encapsulating of chemotherapeutics into EVs was suggested to reverse the drug resistance of cancer cells. Accordingly, one of the studies revealed the highest efficiency of paclitaxel loading into macrophage-derived EVs by mild sonication, when compared with passive incubation and electroporation. In addition, these paclitaxel-loaded EVs induced clinically relevant anti-neoplastic effect in mice with lung metastases [71]. Similarly, EVs from human ovarian cancer SKOV3 cell line can be loaded with triptolide by sonication, which induces their pro-apoptotic effect [72]. Interestingly, sonication may also be effective for loading of therapeutic siRNA, which induces oncogene knockdown [73]. This procedure seems to weakly affect EV quality and, thus, may be considered fast and reproducible for common use [74]. However, the sound energy has to be individually chosen as it may cause EVs’ disintegration [75,76].

#### 3.2.5. Hypotonic Dialysis

Former studies showed that human red blood cells subjected to hypotonic dialysis are capable of encapsulating drugs by diffusion and, to a lesser extent, by endocytosis [77]. This observation might prompt the hypothesis that controlled hypotonic dialysis can be used for encapsulating molecules into EVs. To our best knowledge, this strategy was so far used in one study evaluating the efficacy of porphyrin loading with the use of different methods. Interestingly, in that case, dialysis appeared the most efficient strategy, when analyzing the number of drug molecules acquired by single vesicle [53]. Altogether, these findings revealed the significant potential of hypotonic dialysis as a promising loading strategy with very low impact on EVs’ quantity. However, the possible influence of dialysis on the size and surface charge of EVs was also reported [53].

#### 3.2.6. Freeze-Thaw Cycles

Subjecting EVs to thermal shock through freeze-thaw cycles can be damaging to their membrane, but its precise performing may facilitate the loading process by producing temporary pores [31]. However, the loading efficacy is quite low due to the significant loss of vesicles caused by their disintegration. Additionally, thermal shock affects EVs’ morphology and promotes their aggregation [31,56], which, to some extent, might be prevented by the use of trehalose [78]. One can speculate that thermal shock may impact the physicochemical properties of molecules at the time of loading. On the other hand, freeze-thaw cycles are also used to lyse EVs for extraction of their content [79]. Accordingly, EV content, including RNA and DNA preparations, extracted from thawed EVs was found stable and detectable in various analytical methods [80,81,82,83,84,85].

However, thermal shock was found quite efficient for loading of catalase into RAW 264.7 macrophage-derived EVs [56], and hollow gold nanoparticles into EVs from mouse melanoma B16-F10 cell line [31]. Furthermore, freeze-thaw method was employed to fuse mouse cell line-derived EVs with synthetic liposomes. Cellular uptake of the resulting engineered hybrids could be enhanced by manipulating lipid composition of liposomes [86]. This observation has significant clinical potential in designing EVs for drug delivery. Besides, it seems to support the above hypothesis on the adjuvant role of EV membrane lipids.

#### 3.2.7. Extrusion

At first, extrusion was employed to obtain cell-engineered nanovesicles by serial (e.g., three-fold) extrusion of EV-parental cells through the polymeric (e.g., polycarbonate) filter membranes with diminishing pore size, followed by isolation of resulting vesicles (e.g., by ultracentrifugation) [87,88,89,90]. Subsequently, this method was modified to allow production of drug-loaded EVs. It relies on incubation of parental cells with chemotherapeutic molecules, which is followed by extrusion to produce drug-loaded EVs [91,92]. Other studies also followed different protocol based on incubation of EVs themselves with selected molecules and their further extrusion [53,56]. Regardless of the protocol, extrusion is the most artificial method out of those described, but likely is the most effective in the terms of the quantity of obtained EVs that are loaded with chosen molecules. However, yielded nanovesicles, to some extent, are artificial, which may affect their biocompatibility. Thus, further studies are required to validate this procedure for producing nanovesicle-based carriers.

Along these lines, extrusion method was recently used to fuse EVs with lipids to generate uniform lamellar nanovesicles characterized by high cellular uptake rate. They were also successfully loaded with siRNA by electroporation [93]. These findings provide new insights into the process of engineering highly efficient nanovesicle tool for delivery. In addition, they support the above hypothesis on the adjuvant activity of lipids composing EV membrane.

#### 3.2.8. Novel Approach to Loading of Nucleic Acids

Encapsulation of nucleic acids into EVs is fraught with many challenges since they constitute a very labile cargo, which can easily degrade due to loading conditions. To overcome this problem, recent report proposed protonation of EVs to generate a pH gradient across their membranes as a mean for efficient loading of nucleic acids, especially miRNA, siRNA, and single-stranded DNA. This method takes advantage over other active loading strategies since it does not require the introduction of energy that can damage nucleic acids [94].

### 3.3. Directing EVs towards Desired Target

Loading EVs with selected cargo seems to be crucial for induction of expected biological effect. However, directing EVs towards desired target cells is likely the most important step to achieve the highest efficacy of EV-mediated therapeutic effect. Directed targeting greatly increases the dose of EVs that reach the desired cells and tissues and, simultaneously, limits the unwanted engulfment of EVs by other cells, including phagocytes.

Currently, some researchers attempt to genetically modify the parental cells to facilitate the selective tissue targeting by derived EVs. Along these lines, EVs generated by engineered immature DCs expressed membrane protein (Lamp2b) that was fused to αv integrin-specific iRGD peptide, which mediates tumor homing [95]. Otherwise, EV-parental cells were transfected with plasmid containing cDNA sequence for anti-HER2 antibody single-chain variable fragment (scFv) of ML39 clone, which allowed generation of EVs that expressed the antibody. After in vivo administration into mice with implanted HER2-positive breast tumor, these directed EVs, additionally loaded with mRNA for enzyme that activates chemotherapeutic prodrug, were found most effective [50]. In another study, AS1411 DNA aptamer that binds to nucleolin abundantly expressed on breast cancer cells was used as tumor targeting ligand. Its conjugation to cholesterol in EV membrane ensured selective, tumor cell-targeted EV action [96]. Future perspectives in cell targeting may be based on interactions between receptors and ligands as well as on specific binding of antigen by antibodies.

#### 3.3.1. Approaches Based on Receptor-Ligand Interactions

**Figure 1:**
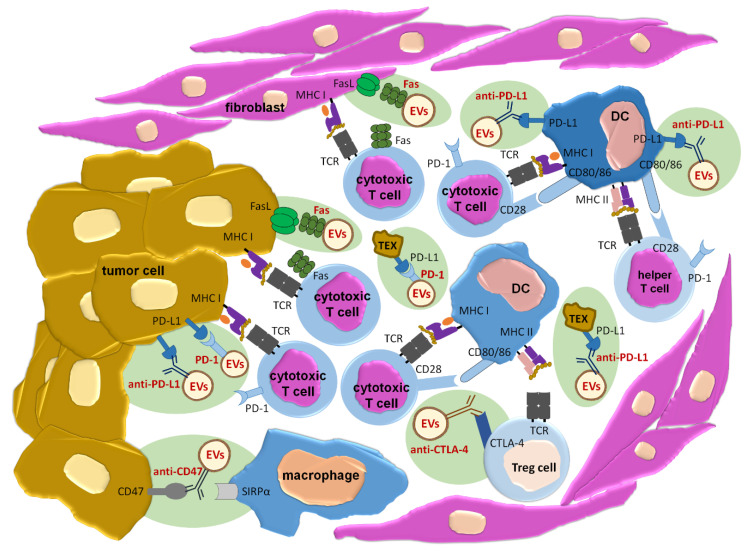
Perspectives in clinical application of extracellular vesicles (EVs) targeting the desired cell through receptor-ligand interaction or specific antibodies in prevention or restoration of immune tolerance to cancer. Sites of possible EVs’ action are additionally marked with a greenish background. anti-—determines the specificity of monoclonal antibodies; CTLA-4—cytotoxic T-lymphocyte-associated protein 4; DC—dendritic cell; FasL—Fas ligand; MHC I—major histocompatibility complex class I; MHC II—major histocompatibility complex class II; PD-1—programmed death receptor-1; PD-L1—programmed death receptor-1 ligand; SIRPα—signal regulatory protein *alpha*; TCR—T cell receptor; Tex—tumor cell-derived extracellular vesicles; Treg cell—T regulatory cell.

Receptor-ligand interaction is an already acknowledged mechanism, by which EVs induce intracellular signaling and, thus, the resulting biological effects [5]. On the other hand, such an interaction involves highly selective binding of two molecules, which may be used for directing EVs towards desired cell. Thus, targeting strategies based on receptor-ligand interaction may provide combined possibility to direct EVs towards particular cell type simultaneously with induction of expected biological effect (Figure 1).

Considering the immune checkpoint therapy, tumor cells often express PD-L1 responsible for induction of cytotoxic T cell exhaustion, and release PD-L1-expressing EVs that can abolish the effect of anti-PD-L1 or anti-PD-1 therapies [7,97]. Genetic blockage of PD-L1 expression on tumor EVs rescued systemic anti-cancer immunity in a mouse model [98]. Since genetic treatment is fraught with many variables and is still difficult to carry out in human patients, one can speculate that administration of EVs equipped with surface PD-1 molecules may efficiently substitute such a mean of treatment. Expression of PD-1 would allow the EV-mediated therapeutic effect by two mechanisms: firstly, by blocking of deleterious activity of tumor PD-L1-positive EVs, and secondly, by directing EVs towards tumor cells to induce their death by contained cargo. Analogous therapeutic effects could be achieved by administering EVs armed with anti-PD-L1 antibodies, as discussed below. Additionally, tolerogenic DCs expressing PD-L1 [99,100] can be targeted by EVs equipped with PD-1 molecules or anti-PD-L1 antibodies.

Some tumor cells express Fas ligand (FasL) molecule, which makes them capable of direct killing of immune cells that infiltrate tumors [101,102]. This constitutes another sophisticated strategy of tumor escape. It can be assumed that this strategy could be efficiently blocked by administration of EVs expressing Fas molecule, which will direct EVs towards FasL-positive tumor cells. Recent report revealed that cancer-associated fibroblasts can process and cross-present tumor antigens to cytotoxic CD8+ T cells in tumor niche. This enables precise killing of antigen-specific cytotoxic T cells in FasL-dependent manner [103]. Thus, Fas-expressing EVs may also target fibroblasts and other cell types in tumor microenvironment to reverse immune response suppression in a wide range.

Apart from EV directing, receptor-ligand interactions may supposedly trigger the expected biological effects in targeted cells. For instance, tumor necrosis factor (TNF)-related apoptosis-inducing ligand (TRAIL)-expressing EVs may potentially activate apoptosis in targeted tumor cells [104]. Similar pro-apoptotic effect can be induced by FasL-expressing EVs. However, Fas expression on tumor cells is usually significantly reduced [105]. Therefore, EV-mediated therapeutic effects may be greatly enhanced by EV-contained cargo that would be selectively delivered to acceptor cell.

The main challenge of the use of receptor-ligand interaction for EV trafficking concerns the need to select such a molecule, i.e., receptor or ligand, that is overexpressed on desired cell subtype, but is weakly expressed by other cells of the body.

#### 3.3.2. Approaches Based on Antigen-Specific Interactions

As peptides with major histocompatibility complex (MHC) can be expressed by antigen-presenting cell-derived EVs [106], they are also the first molecules to consider for targeting antigen-specific T cells. Conversely, T cell receptor (TCR)-bearing EVs could be proposed for targeting antigen-presenting cells. However, interaction of specific antibodies or their light chains with antigenic determinant seems to be the best suited for directing EVs in the most precise manner. To avoid non-specific cell targeting, a specific antigen present mostly on desired cells has to be identified.

Along these lines, liposomes were recently proposed to constitute a promising carrier for intracellular trafficking of therapeutic antibodies against molecules located within the cell [107]. On the contrary, cell targeting by liposomes and other synthetic nanoparticles can be conferred by functionalizing their surface with specific antibodies [108]. Analogously, targeted EVs can be generated by displaying specific antibodies or their fragments on EV surface membrane [109].

Our studies revealed the presence of antigen-specific antibody *kappa* LCs on the surface of EVs released by mouse suppressor T cells, which allowed us to separate antigen-binding EV sub-population, as mentioned above [40]. Further research uncovered that LCs confer the specificity of cell targeting by EVs [110,111]. In addition, LCs may naturally coat EV surface in circulation or in cell culture medium [40,110], but can also be in vitro coated onto EV membrane by simple incubation [42]. Our initial data suggest that LC binding is mediated by membrane lipids [111,112].

As mentioned above, PD-1/PD-L1 checkpoint activities can be modulated by EVs equipped with either PD-1 molecules or anti-PD-L1 antibodies. While antigen–antibody interaction is highly specific due to the extremely high affinity of the binding, receptor–ligand interaction is considered selective and the binding strength, to some extent, may be more sensitive to current microenvironmental conditions. Thus, one can speculate that the use of antibodies may greatly enhance the therapeutic effect. Furthermore, tumor cells can escape the anti-cancer immunity by activating regulatory T lymphocytes that express cytotoxic T-lymphocyte-associated protein 4 (CTLA-4) competing with CD28 of effector T lymphocytes for binding to CD80/CD86 of antigen presenting cells [113]. This phenomenon, called clonal anergy, leads to suppression of anti-tumor, cytotoxic, and helper T cell-mediated immune responses. However, this tolerogenic effect could be reversed by the use of EVs expressing anti-CTLA-4 antibodies. Altogether, the therapeutic administration of EVs equipped with antibodies that provide the blockage of immune checkpoint molecules is a promising strategy to avoid or reverse patient resistance to cancer treatment [114,115].

Recent findings implied the role of cancer cell-expressed CD47 in inhibition of anti-tumor immune responses. Namely, stimulation of the CD47/ signal regulatory protein *alpha* (SIRPα) axis abolishes phagocytosis of malignant cells and macrophage cytotoxicity against tumor cells. Therefore, therapeutic anti-CD47 antibodies for blockage of “don’t eat me” signal are under investigation [116]. Supposedly, displaying these antibodies onto EV membrane may both facilitate tumor targeting and increase the dose of antibodies that bind each cell.

Obviously, therapeutic effects of antibody-directed EVs would be then induced by EV-contained cargo. It is also worth noting that antibodies and LCs are easily biodegradable by proteases. Thereby, they may be considered as physiological targeting mediators (Figure 1). Furthermore, the use of antigen-specific antibodies or LCs greatly increases the specificity of tissue targeting by EVs [117].

### 3.4. Selecting the Optimal Route of EVs’ Administration

Depending on the route of administration, antigens may be either immunogenic or tolerogenic [118]. Analogously, one can speculate that the route of EVs’ administration may either increase or diminish their eventual effect. Furthermore, it also determines the biodistribution and bioavailability of EVs as well as may facilitate their barrier-crossing ability. Thus, delivery route is one of the essential factors determining the overall efficiency of EVs’ therapeutic activity [119]. On the other hand, route of therapeutic EVs’ administration should be accepted by patients.

So far, various routes of EVs’ administration have been experimentally examined. Some showed that intravenous route is more efficient than intraperitoneal injection [120], and that intradermal application has an advantage over subcutaneous treatment [121]. Interestingly, intravenously infused EVs were shown to co-localize with microglia in injured spinal cord of contused rats [122]. Furthermore, intranasally administered EVs can be incorporated by neurons and microglia [123]. Moreover, orally administered EVs from bovine milk were found to ameliorate arthritis in mice [124]. Similarly, we have observed that EVs released by suppressor T cells from mice tolerized to casein, suppress casein-induced delayed-type hypersensitivity response after administration via intravenous, intraperitoneal, intradermal and oral routes into actively immunized mice [41]. Several other studies also suggested the functional activity of EVs delivered via oral route [125,126]. Therefore, oral route of treatment seems to be promising approach, firstly due to its accessibility and well acceptance by patients, and secondly, as it is amenable for repetitions. However, EVs’ formulations and dosing protocols for oral treatment must be well established to avoid variability in therapeutic efficacy.

## 4. Conclusions

The goal of this review was to comprehensively discuss the knowledge on currently available methods as well as future perspectives in manipulating EVs for therapeutic applications with a special emphasis on cancer treatment. EVs’ biology and their clinical applications are tremendously complex research areas. We hope that this review provides some useful insights for possible strategies and innovation in EVs’ applications, while being aware of the virtually inexhaustible nature of the undertaken topic [127]. Extracellular vesicle research can be compared to exploring a newly discovered cave. The deeper you enter the cave, the more new side corridors you will find for exploration.

## Figures and Tables

**Table 1:** Comparison between the methods used for loading Extracellular vesicles (EVs) with selected cargo.

Method	Principle	Advantages	Disadvantages
passive loading	joint incubation (sometimes in special media or buffers)	simplicity and very low impact on EVs’ and cargo quality	unpredictable efficacy
electroporation	transient formation of pores in EV membrane with electrical pulse	high efficacy for RNA loading	very high impact on EVs’ quality and quantity ^1^
treatment with saponins	detergent-induced permeabilization of EVs’ membrane	high efficacy and low impact on EVs’ quality	impact on loaded cargo ^2^
Sonication	sound energy-induced agitation facilitating incorporation of molecules by EVs	simplicity, good efficacy and low impact on EVs’ quality	the need for individual standardization of the protocol
hypotonic dialysis	subjecting the mixture of EVs and cargo to dialysis in hypotonic conditions	simplicity	possible impact on EVs’ quality
freeze-thaw cycles	thermal shock-induced transient formation of pores in EV membrane	simplicity and good efficacy	very high impact on EVs’ quality and quantity
extrusion	serial extrusion of drug-pre-incubated EV-parental cells through the polymeric filter membranes with diminishing pore size	very high quantity of yielded EVs	artificial generation of EVs

^1^ Chemical transfection methods may help to overcome these disadvantages. ^2^ Can be used only for encapsulating of detergent-insensitive molecules.

## Abbreviations

CTLA-4	Cytotoxic T-lymphocyte-associated protein 4
DCs	Dendritic cells
EVs	Extracellular vesicles
FasL	Fas ligand
LCs	Light chains
MHC	Major histocompatibility complex
miRNA	Micro ribonucleic acid
PD-1	Programmed death receptor-1
PD-L1	Programmed death receptor-1 ligand
siRNA	Small interfering ribonucleic acid
SIRPα	Signal regulatory protein alpha
